# The Bread We Eat

**Published:** 1855-12

**Authors:** 


### HALL'S JOURNAL OF HEALTH.

OUR LEGITIMATE SCOPE IS ALMOST BOUNDLESS: FOR WHATEVER BEGETS PLEASURABLE AND HARMLESS FEELINGS, PROMOTES HEALTH; AND WHATEVER INDUCES DISAGREEABLE SENSATIONS, ENGENDERS DISEASE.

VOL. II.] DECEMBER, 1855. [NO. XII.

### THE BREAD WE EAT.

If anybody can visit Mr. Pease's *Five Points Mission* at 11 A.M. any Sunday, and remain until some hundred little orphan children of all sizes and colors gather around a long table, and each with a bowl and spoon takes the broth or pudding which charity has provided, and without which many of these helpless creatures would have gone without a dinner or a supper, and some perhaps, yes certainly, would have done worse, if anybody can witness such a sight, or can read the history of dying little *Tommy*, as recorded in the *Five Points Monthly* for May, and not feel that wonderful chill of emotion which precedes a tear, it is more than we can do. If, under such influences, any one can help wishing he was as rich as Astor or Longworth, that he might give it all away to happify the outcast and friendless children of poverty and crime of all lands, such an one must be made of sterner stuff than we are. But, reader, it is not by the largesses of the millionaire, so much as by the mite of the widow, that humanity is to be raised to heaven. There are but few millionaires, there are countless myriads who can afford to give but a few dollars, and in this great New York, there are scores of thousands who can afford to give only cents.

This article is written to present a plan by which the receipts of "the Mission" may be doubled by the moneyless wives and daughters of New York City, without asking their husbands or fathers for a single penny, without begging it from anybody, but by earning it themselves creditably and independently, then comes back to us "into the bargain" that sweetness and that force, which is involved in every kind act which costs us something. What good can ever come to that wife or daughter's heart who takes the dollar from the husband's or father's hand, and passes it in the poor-box! This automatic charity is not worth a pinch of dust; for a pinch of dust even, would manure a flower-seed, to spring up and bless with its freshness and its beauty a hundred admiring eyes, and yet have all that freshness and beauty undiminished still, to happify just as many more; but down into the heart of a tool of charity, there sinks not one fructifying atom, no soul-blessing aroma.

But let us descend to "Bread," for we generally come back to the text, if we do not "*stick*" to it. Let every wife and daughter in New York go to Stringer & Townsend's, 222 Broadway, or No. 1 Vesey Street, under the Astor House, and purchase Hall's "Journal of Health for June" for ten cents, and besides learning the value of *money as a medicine—How to leave church—How to be eloquent—How to use fruits—How to avoid sickness in summer*, and other useful things, they will learn at page 148, how to bake a better, more nutritious and more healthful, because purer bread, than any New York baker ever served to his customers. Practice a little on this, meanwhile make a note of how long it requires to spend the price of a barrel of flour at the baker's, say thirteen dollars fifty cents, that being the cost of the last "*Extra Genesee*" furnished us by Messrs. Hecker of Cherry Street, but that "Genesee" ever saw said flour, no one for a moment imagines, or that "Genesee" ever grew the wheat out of which it was made, is one of the "fictions" of trade, in the opinion of the Gnostics. But for all that, these gentlemen do furnish us the whitest and best flour in the market, and as the most certain way of obtaining the best manufactured article of bread is to have the best material, we advise the purchase from these gentlemen, or some other of the best and most reliable flour dealers.

Now throw together the following items: you pay your baker ten dollars a month, a hundred and twenty dollars a year, and as flour is falling, call a barrel twelve dollars.

A baker averages two hundred and sixty-five pounds of bread from each barrel of flour, and you pay for it at the rate of near seven cents a pound, for a New York loaf costs 6¼ cents, and I have not lately seen one that weighed a good pound; then, suppose you get your flour at twelve dollars a barrel, that same barrel would make you two hundred and sixtyfive pounds of bread, or would yield you eighteen dollars and fifty-five cents worth, at bakers' prices, giving you a clear saving of six dollars fifty-five cents on each barrel; and this, at the rate of a barrel of flour a month, would be a saving of over seventy-eight dollars a year, to be paid to prevent helpless and forsaken children from falling into starvation and crime, instead of being paid to bakers, guiltless of ever having sold a single loaf of pure bread, made from flour A. No. 1.

There is scarcely a family in New York which does not consume *two* barrels of flour a year, averaging ten dollars a barrel, for persons of moderate means do not usually purchase the best brands; and even at this figure of two barrels a year, there would be a saving of over seventeen dollars a year, which a family of very moderate means could thus bestow in charity annually, and not be a dollar the poorer; for the time spent in baking this bread, would require exercise, which would result in health and strength and ability to perform an amount of labor in other departments of the family, largely remunerative, above what it would otherwise be.

The only actual loss would be the extra fuel; but where a fire has to be made in the kitchen daily, anyhow, the "little more" necessary to bake bread, would be trifling, and with a proper supervision over the cook, would be almost nothing.

A considerable per centage of some bakers' bread is made out of potatoes, costing at most a cent a pound, and sold in the shape of bread, at six cents a loaf, yields an advance of three hundred per cent. The alum used to whiten the bread is sold at an equal advance.

There is a phrase at the "Corn Exchange" of some meaning to wit, *Sour Flour*, of which bakers are liberal purchasers. This includes flour which has become musty, the sunken in boats and ships and soaked for days, weeks, or months in bilge water, this is mixed with *good* or *fair* flour, never the prime article, and delivered to us in the shape of pound loaves, weighing just thirteen ounces, that being the weight of the loaves at the bakers nearest to us at the present writing. This "Sour Flour" costs from one to four cents a pound, the alum and the potatoes in it about the same, making an average of three cents, and is sold at six and a quarter cents for thirteen-ounce loaves. Remembering that each barrel yields a baker two hundred and sixty-five *full* pounds of bread, we arrive at the important fact, that the bakers' bread of New York costs its consumers more than one hundred per cent. above its actual worth: that multitudes in this city, for bread made out of potatoes, alum, saleratus and sour flour, averaging three cents a pound, pay the sum of six and a quarter cents a loaf, which in any case does not contain over fourteen ounces of actual flour, costing the baker just two and a half cents.

It would be wrong to charge that all New York bakers practice these frauds to such extent, but that any baker here, sells for six and a quarter cents, a loaf of bread which contains sixteen ounces of pure flour of A. No. one quality, or ten ounces either, is one of those "fictions of trade" which are so largely dealt in now-a-days.

Since writing the above, a correspondent of the *Daily Times* comes to our aid in the following statement:

"For years I, like most citizens, had depended upon bakers for bread, leaving them to give as large or small a loaf as they pleased for a sixpence, and consuming about eight loaves per day. My baker's bill used to reach $16 for bread, $5 for cake, and $1 for flour for making pies per month, being $22 for provisions made of flour. Hence I determined to try an experiment. I ordered a barrel of flour, which I got for $10, and requested my wife to keep a correct account of the expense of preparing and baking said barrel of flour into bread, cake and pies, for the supply of my family. She did so, and the results were as follows: One barrel of flour, $10; yeast, 25 cents; sugar and eggs for cake, $1 38; paid for baking, $2 62—making $14 25, for what it cost when brought from the baker's, $22; thus making a difference in my favor of $7 75 between buying the flour and manufacturing it to suit us, ourselves, and depending upon bakers. The barrel of flour lasted just one month for bread, cakes and pies."

If this practical gentleman were to say to his wife, "Let us take this saving of seven dollars and three-quarters a month, or ninety-three dollars a year, and appropriate it to the objects of the '*Five Points Mission*,'" can any one doubt but that the world would be left the better for their having lived in it, or that they themselves would sink more sweetly into the sleep which knows no waking?

From an accurate calculation, it can be shown that when flour sells at six dollars a barrel, the baker can sell his bread at three cents a pound and make a clear profit of fifty per cent.; if he pays twelve dollars a barrel, he can sell one pound loaves at six cents, and clear fifty per cent.

In view of these statements, we may well inquire:

Ought we to patronize the fraudulent? Would we not do humanity a service by practising the economies suggested in this article?

Failing to do so, is a sin of omission, less than one of commission?

Would any man in Fifth Avenue, or Fourteenth Street, or Union Square, look less admiringly on a daughter, or less lovingly on a wife, whom he should detect in economies like these, and for the objects named.

Is any young woman living, less fit for marriage, from knowing how to bake a loaf of bread? Will she be less loved or valued, ten years after marriage, by the man who is to be her husband?

Would this schooling of her industry, of her calculation, of her principle, of her charities, make her any the less a woman of character, of influence and of power, when she becomes the mistress of her own mansion?

These things should be pondered well. The homeless orphan has a strong claim on the big heart of humanity. Whether its parentage was that of improvidence, of misfortune or of crime, it stands before us as "*The Friendless Innocent,*" and to save it from starvation now, and crime hereafter, and to put it in the way of being useful and happy in mature life, *by the work of our own hands*, by that which costs us either labor or self-denial, is such a mission as any noble heart might well be proud of; it is a mission to "*the Greek at the door;*" and in a more ennobling one, the wives and daughters of New York cannot engage. Will you do it, my countrywomen? If not, Humanity asks to know the reason why. Let it be such an one as you will not be ashamed to avow at the judgment of the great day.

But as to the "*Cui Bono,*" *what's the use* of sending our wives and daughters to the kitchen to bake bread, instead of paying the baker for it, and then to give the "*savings*" all away to poor people's children? or what is worse, to the children of

"*nobody's*," and vagrants and criminals? The noblest and most convincing answer we can give, is contained in an advertisement in the NEW YORK HERALD for Sept. 9th, 1855, which reads as follows:

"FIVE POINTS MISSION.—Rev. Mr. Van Meter, agent of the Ladies' Mission at the Five Points, who recently took twenty-eight destitute children to Illinois, where he has placed each in a good home, is now in this city for the purpose of obtaining additional children, for whom excellent homes have been secured. Mr. V. M. will return to the West in a few days. A public meeting will be held this evening at half past 7, at which time Mr. Van Meter will present a number of interesting facts in relation to the West, the children he has taken there, and the homes provided for them."

Well done, Mr. *Van Meter;* May God's help go with you, and the orphan's blessing attend you to life's latest hour.

Or, in case some families in good circumstances should prefer to give a different direction to this savings fund, such may embalm its name in sweet memoriesfor all time, by taking up and caring for one of the two hundred orphan-stricken little ones whom the yellow fever at Norfolk has deprived of father and mother and home and kindred, and who, unless some kind heart takes them up, must still remain lost, lonely, and forsaken!

